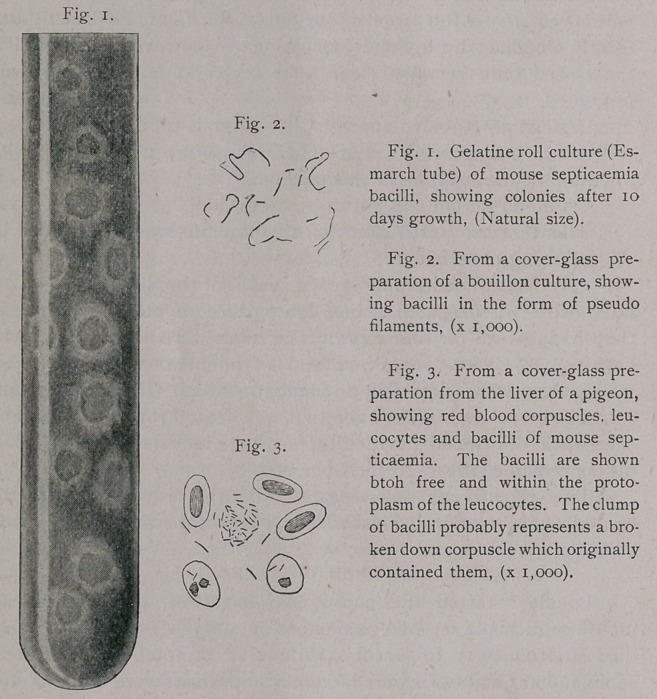# Mouse Septicæmia Bacilli in a Pig’s Spleen

**Published:** 1892-06

**Authors:** Veranus A. Moore

**Affiliations:** The Division of Animal Pathology, Bureau of Animal Industry, Dapartment of Agriculture, Washington, D. C.


THE JOURNAL
OF
COMPARATIVE MEDICINE AND
VETERINARY ARCHIVES.

VOL. XIII. JUNE, 1892. No. 6.

### MOUSE SEPTICÆMIA BACILLI IN A PIG'S SPLEEN, WITH SOME OBSERVATIONS ON THEIR PATHOGENIC PROPERTIES

By VERANUS A. MOORE, B. S., M. D., of the Division of Animal Pathology, Bureau of Animal Industry, Dapartment of Agriculture, Washington, D. C.

The similarity of the bacilli of mouse septicæmia to those of *rouget* (swine erysipelas) has already been pointed out by Loeffler * who demonstrated, experimentally, the resemblance which exists between these bacteria in their microscopic appearances, their growth in gelatine, and their behavior towards the white blood corpuscles in the intfected animal. This, together with the fact that the bacillus of *rouget* is the cause of a very important swine disease in Europe, while the other is a septic germ found in decomposing albuminous substances, and fatal only to certain of the smaller experimental animals, renders its appearance in tissues that have not undergone putrefactive changes to any appreciable degree a matter of considerable interest.

In the bacteriological investigations of decomposed animal substances, Koch † found that mice inoculated with from one to two drops of putrid blood would die in from forty to sixty hours, and upon examination, their blood, spleen and liver were found to contain a very large number of slender bacilli, either single, or lying in clumps between the blood corpuscles. The blood of the mice, dead from these inoculations, was found to be fatal to other mice, when as small a quantity as 0.1 of a drop was injected beneath

* *Loeffler.*—Arbeiten a. d. Kaiserlichen Gesundheitsamte, Berlin. Bd. I. 1885, p. 47.
† *Koch.*—Untersuchungen uber die Aetiologie der Wundinfectionskrankheiten, 1878, p.

the skin. Koch carried on such a series of inoculations, through fifty-four mice, and always with the same fatal results. He described the bacillus as a rod-shaped germ, measuring from 0.8 to 1.0 $\mu$. in length, and its thickness so slight that an exact measurement could be made, but approximately it was about 0.1 $\mu$.

Other European investigators have found this bacillus in material similar to that in which Koch first discovered it, and several additional facts have been recorded concerning its morphology and pathogenic properties.

Flügge * states that sparrows and pigeons are susceptible, and that rabbits sometimes die from inoculation of this germ, but usually only a local affection is produced. He describes its growth in gelatine as a bluish-gray chain ; the thickness of the bacillus as 0.2 $\mu$. Cornil and Babes † described the bacillus as being 2.0 $\mu$. in length.

In the investigation of animal diseases carried on by the Bureau of Animal Industry, this bacillus has been found on two different occasions in pigs that were examiaed in a fresh condition. So far as I can learn, this is the first record of its appearance, excepting in decomposed animal matter.

*First Appearance.*—In the investigation of the cause of swine plague (hog cholera) in 1885, Dr. Theobald Smith found the bacillus of mouse septicæmia in the kidney of a pig ‡ that was killed in the last stages of disease. A description of this bacillus was published § at that time, although no name was assigned to it. The description of its microscopic appearance, its method of growth in gelatine, and its effect on mice are sufficient, however, to identify it, and to differentiate it from the bacillus of *rouget.* Although Dr. Smith published nothing further concerning this germ, he studied it very carefully subsequently, and identified it beyond the possibility of a doubt as Koch's bacillus of *mouse septicæmia.*

*Second Appearance.*—October 3, 1888, I made a bacteriological examination of the spleen of a pig that died at the Experiment Station of the Bureau. It was removed from the animal under strict antiseptic precautions, placed in a sterilized bottle, and brought to the laboratory. It was examined at once. The surface was scorched, and from the pulp underneath a tube of bouillon was

---

* *Flugge.*—Die Mikroorganismen, 1886, p. 250.

† *Cornil et Babes.*—Les Bacteries. 1890, p. 270.

‡ Dr. Smith tells me that there was an invasion of the peritoneal cavity and the various organs of this pig with bacteria from the intestinal tract, and that they most likely obtained entrance through the numerous and extensive necroses of the mucous membrane.

§ The Second Annual Report of the Bureau of Animal Industry, 1885, p. 196.

inoculated and a gelatine roll culture made. A stained cover-glass preparation from the tissue exhibited no bacteria. The gelatine roll remained clear. The bouillon was clouded the following morning, and upon a microscopical examination (hanging drop) was found to contain two species of bacteria, one motile, and the other non-motile. The motile bacillus proved, upon further investigation, to be the bacillus of hog cholera. The other bacillus was very long and slender, and presented the appearance of the bacillus of mouse septicæmia.

Oct. 6, two mice were inoculated subcutaneously with a very small quantity of the bouillon culture. These were found dead the mornings of the second and third days Stained cover glass preparations from the various organs of these mice revealed the presence of a very large number of long, very slender bacilli. Agar and gelatine tubes inoculated from the blood gave pure cultures of this bacillus which developed in the gelatine the characteristic growth of mouse septicæmia. An agar culture from the spleen of mouse No. 1 contained motile hog-cholera bacteria. No culture was made from the spleen of mouse No. 2.

Oct. 12. A series of gelatine roll cultures were made from the original bouillon culture. These developed many colonies of hog-cholera and a very few colonies of mouse septicæmia bacilli.

Oct. 16. Two mice were inoculated with 3-5 drops of the original bouillon culture. These died on the second and fourth days respectively and from each only hog-cholera bacteria were obtained.

From these experiments the conclusion seems warranted that the original bouillon culture from the pig's spleen contained both hog-cholera and mouse septicæmia bacilli, and that in the first inoculation the very small quantity of virus used was sufficient for the mouse septicæmia to destroy the animal before the very few hog-cholera bacteria that were introduced could become disseminated throughout the body. This is indicated by their non-appearance in the cultures from the blood. The age of the culture at the time of the last inoculation may have been sufficient for the specticæmia germs to have perished, leaving only the hog-cholera bacteria which destroyed the mice.

### MORPHOLOGY AND BIOLOGY.

*Morphology.*—Slender non-motile bacilli appearing as short rods or long more or less tortuous filaments. In bouillon cultures the short rods are about 2 $\mu$. long and 0.4 $\mu$. thick. The filaments vary from 4.0 to 15.0 $\mu$. in length. In cover-glass preparations from the liver and spleen of pigeons the bacilli appear in larger or smaller clumps lying between the cells. They are occasionally seen enclosed within the cellular elements, especially the leucocytes. They are stained readily with the aniline dyes. They take the Gram stain.

*Agar at* 35° *C.*—On the surface of agar there appear after twenty-four hours round, translucent, convex colonies varying from ⅓ to ½ $\mu\mu$. in diameter. In the condensation water a very slight growth. By the third day the colonies, if not crowded, will increase to 1 $\mu\mu$. in diameter; they are round, grayish, with a glistening surface and sharply-defined border. A slightly viscid deposit in the condensation water. Within agar minute grayish colonies are developed. On glycerine agar the growth does not differ appreciably from that on the simple agar.

*Gelatine.*—In needle cultures after twenty-four to thirty-six hours at the ordinary temperature there develops along the needle track a quite dense, grayish line, due to the crowding together of minute colonies. From the growth along the track of the needle faint, cloudlike processes extend laterally almost to the sides of the tube, giving the gelatine a clouded appearance, and to the growth a form resembling that of a "test tube brush." The gelatine is softened, but rarely liquefied. In gelatine rolls no colonies are visible after twenty-four hours. On the second day the colonies, when not crowded, appear in reflected light as bluish-gray nebulous spots; by transmitted light they appear more distinctly as very delicate, bluish translucent areas. Under a low-power lens nothing can be seen. The colonies in general are round, but they have no sharp outline, gradually fading into the surrounding clear gelatine. After six days the colonies attain a diameter of 4–7 millimeters, with round, slightly more opaque centers, a few of which appear to be quite solid. Several days later the colonies are very indistinct and cloudlike. There is a small amount of liquefaction of the gelatine which does not seem, however, to be associated with the colonies. When crowded they present the appearance of two kinds of colonies, (1) those which have a simple nebulous appearance and (2) those which have a minute solid nucleus. The latter are much smaller than the former. The largest are about 2 $\mu\mu$. in diameter. They are separated by a distance of ¼ to ½cm. At the end of ten days about ½ of the colonies show the solid nucleus. The colonies are about ½ to 1 $\mu\mu$. in diameter and consist of a central opaque portion surrounded by a translucent band which is bounded externally by an opaque ring.

*Peptonized buillon at* 36° *C.*—The day following its inoculation the liquid is faintly but uniformly cloudy. When the tube is shaken this cloudiness has the appearance of clouds of dust. A few days later it is less marked with a very slight deposit in the bottom of the tube.

*Peptonized bouillon containing* 2 *per cent. glucose in fermentation tubes at* 36° *C.*—After twenty-four hours the liquid is usually uniformly clouded throughout. Occasionally, however, the liquid in the closed bulb remains clear for forty-eight hours. Gas is not produced.

*Milk at* 36° *C.*—No appreciable change is produced in its appearance. A microscopical examination shows a vigorous multiplication of the bacteria in this medium.

On blood serum and potatoes, no growth.

The thermal death point of this bacillus was determined by the following experiment:

Five tubes containing 10 cc. each of bouillon were inoculated with 1-2 drops of a bouillon culture of mouse septicæmia that had grown for four days. The tubes were heated in a water bath at 58° C. for 5, 10, 15, 20 and 25 minutes respectively, after which they were placed in an incubator at 36° C. A check tube was also inoculated. The tube that was heated for 10 minutes and the check developed pure cultures of the inoculated germ. The others remained clear.

This experiment was repeated with the result that all of the heated tubes remained clear. The check tube developed a pure culture.

### PATHOGENIC VALUE.

Loeffler* tested the pathogenic effect of the bacillus of mouse septicæmia on a large number of animals. Mice invariably died in from forty to seventy-two hours. Amphibians (frogs and salamanders) and fowls were immune. Sparrows were quite as susceptible as mice. A large male pigeon inoculated subcutaneously over the pectorals died in eight days. In the blood of these animals a very large number of bacilli were found lying between the cells or enclosed within the white blood corpuscles. Dogs, cats, guinea-pigs and white rats exhibited more or less local reaction. In rabbits the local effect was very marked. An interesting observation was the frequent reappearance of the disease in the opposite ear several days after the reddening about the place of inoculation had disappeared. Loeffler also found that in all cases where there

* *Loeffler.*— Mittheilungen a. d. Kaiserlichen Gesundheitsamte I, 1881, p. 134.

was a marked local reaction in rabbits that a subsequent inoculation after the expiration of a certain period was negative in its results. The length of time required to destroy a pigeon and the very marked effect upon rabbits would indicate a slight difference in the virulence of the germ used by Loeffler and the one we have studied.

In the inoculation experiments that I have made with pure cultures, the germ isolated in 1888 was employed. In the first mice inoculated with the impure culture there was a considerable sanguinolent effusion in the subcutis at the point of innoculation. This was the case with some of the mice inoculated by Dr. Smith in 1885.

*Mice.*—Dec. 10, 1888, a mouse was inoculated subcutaneously with 4–5 drops of a bullion culture. The immediate effect was very marked. Within a few hours the fur became ruffled, and the animal appeared to be somewhat stupified. It moved slowly and apparently with difficulty when touched; respiration irregular and jerky; eyes partially closed, but clear. Mouse was found dead the following morning. The hind limbs were drawn up underneath the body as in the sitting position and the fore limbs extending forward. No effusion of serum at the place of innoculation. Large numbers of slender bacilli observed in stained cover-glass preparations from the spleen, usually in clumps, and frequently within the cells. A smaller number seen in a similar preparation from the blood.

Dec 28. Two mice were inoculated with a bit of blood from a pigeon that perished from mouse septicæmia. They were found dead on the mornings of the third and fourth days, respectively. Enormous numbers of bacilli in spleen and liver, a smaller number in the blood.

*Pigeons.*—Dec. 7, 1888. A pigeon received beneath the skin over the pectoral muscle 1 cc. of a boullion culture two days old. It was found dead on the morning of Dec. 10. No lesions at place of inoculation. Liver pale, sprinkled with areas in which the blood-vessels were much injected. Many bacilli in the liver, comparatively few in the blood. A second pigeon inoculated with 0.5 cc. of the same culture died on the fourth day At the point of inoculation the muscle was very pale, no œdema. Liver hyperæmic. Heart, auricles filled with very dark clots, a small quantity of blood serum in ventricles. Enormous number of slender bacilli in spleen and liver, a small number in the blood. A series of inoculations was made in pigeons, the first inoculated with a bouillon culture, the second from the blood of the first, the third from the blood of the second, and so on, in order to determine the effect of such a series upon the virulence of the germ. The inoculations were made with the point of a lancet. The appended table gives all necessary information concerning this experiment.

| No. of pigeon. | Date of inoculation. | Virus used. | Date of death. | Time required to destroy pigeons. | Remarks. |
|---|---|---|---|---|---|
| 1 | Dec. 7, '88 | ½ cc. Buoillon Culture. | Dec. 10, '88 | 3½ days | Slight local rcaction. many bacilli in spleen and liver, few in blood. |
| 2 | " 10, " | Blood of Pigeon 1. | " 13, " | 2½ " | " " " |
| 3 | " 13, " | " " 2 | " 16, " | 2½ " | No " " |
| 4 | " 17, " | " " 3 | " 21, " | 3½ " | " " " |
| 5 | " 21, " | " " 4 | " 24, " | 2¾ " | Slight " " |
| 6 | " 24, " | " " 5 | " 27, " | 2½ " | Much " " |

It should be added that in all the pigeons there was considerable serum in the pericardial sac, and the amount of hyperæmia varied somewhat in the different organs. It will be observed that the virulence in this short series remained practically constant.

*Other Animals.*—A rabbit and a guinea pig inoculated subcucutaneously with ½ cc. each of a bouillon culture, remained apparently well. A pig was inoculated with 10 cc. (5 cc. in each thigh) on January 7, 1889. January 8, temperature 104.2° F. January 9, temperature 102.1° F. No other symptoms.

The bacillus was kept by means of sub-cultures until the spring of 1890, when a further test of its pathogenic powers was made. A pigeon was inoculated subcutaneously over the pectoral muscle with 0.75 cc. of a bouillon culture. It was found dead in the morning of the third day. The post-mortem examination showed no lesions different from those previously described. A second pigeon inoculated with a small quantity of the liver tissue of this pigeon remained perfectly well. Ten days later it was reinoculated with 0.5 cc. of a bouillon culture of the bacillus, with negative results. It was subsequently killed, and upon examination no lesions could be detected that were produced by the inoculations.

Two mice were inoculated, each with several drops of a bouillon culture, but with negative results. The experiment was repeated with the same result. It would appear that the long cultivation of the bacillus had lessened to a very marked degree its pathogenic properties. Its morphological and cultural characters were not changed to any appreciable degree.

It may be of interest to note a few comparisons that were made of the bacilli of *rouget* with those of mouse septicæmia in parallel cultures and their micscropic appearances. Inoculation tests were not trustworthy, owing to the fact that the culture of *rouget** at my disposal had been under cultivation for about three years, and had become somewhat attenuated. There seemed to be no change, however, in its morphology or culture characters.

Morphologically, the bacilli of mouse septicæmia were about one and one-half times as thick as those of rouget, the filaments, on the other hand, were much shorter and less tortuous. On agar there was no appreciable difference in the character of the growth; the colonies were slightly smaller in cultures of rouget. The rouget bacilli did not develop in glycerine-agar. In galatine needle cul-

* The germ used was obtained from a tube of "vaccine of *rouget*" that was received directly from Pasteur by this laboratory in 1885. In the report of the Bureau for that year, p. 187, is a full account of the experiments made with the vaccine immediately after its arrival.

tures the growth was more dense, and not so diffuse as that of mouse septicæmia. In roll cultures the colonies of rouget bacilli consisted of a central opaque nucleus with a hazy periphery, the whole about 3 $\mu\mu$. in diameter, when the colonies are not crowded, and situated in a liquified area of gelatine extending slightly beyond the border of the colonies The development in roll cultures is especially interesting, as the solid nucleus was observed in a considerable number of colonies of mouse septicæmia. Although no comparative inoculations were made, the earlier investigations with the bacilli of *rouget* showed that they possessed less virulence, for mice at least, than the mouse septicæmia bacilli. There seemed to be a marked tendency on the part of both of these organisms to invade the white blood corpuscles in the inoculated animals.

Although European investigators appear to find the bacillus of mouse septicæmia very readily, I have thus far been unable to discover this germ in decomposed animal tissue The method I employed was to allow the blood, or the solid organs of pigs and rabbits to decompose in the atmosphere, and when the stage of putrefaction was reached mice were inoculated. In a considerable number of such inoculations only negative results were obtained.

With our present knowledge concerning the variability of disease germs, it is of interest to note that in Europe where the bacillus of mouse septicæmia appears to be quite common, *rouget* is prevalent, and that, in this country which, so far as known, is free from *rouget* the bacillus of mouse septicæmia is rarely fonnd. Dr. Smith * has already pointed out variations in the morphology, biology and virulence of the hog-cholera germ that are quite as marked as those apparently existing between the bacilli of mouse septicæmia and those of *rouget*.

In studying the biology of this bacillus and its effect upon animals I have received many suggestions from Dr. Theobald Smith, to whom I am still further indebted for information concerning the bacillus of mouse septicæamia discovered by him in 1885. For the inoculation of the pig and its careful watching subsequently I am indebted to Dr. F. L. Kilborne, Director of the Experiment Station.

---

* *Smith.*—New York Medical Journal, LII. (1890), p. 485.

## Figures and Tables

**Fig. 1. Fig. 2. Fig. 3. f1:**